# N-acetylcysteine treatment following spinal cord trauma reduces neural tissue damage and improves locomotor function in mice

**DOI:** 10.3892/mmr.2015.3390

**Published:** 2015-02-26

**Authors:** JIAN GUO, YIQIAO LI, ZHONG CHEN, ZHENNIAN HE, BIN ZHANG, YONGHUAN LI, JIANGHUA HU, MINGYUAN HAN, YUANLIN XU, YONGFU LI

**Affiliations:** 1 Department of Orthopaedic Surgery, NingboBeilun People Hospital, Ningbo, Zhejiang 315800, P.R. China; 2 Central Laboratory, NingboBeilun People Hospital, Ningbo, Zhejiang 315800, P.R. China; 3 Department of Orthopaedic Surgery, The First Affiliated Hospital of Zhejiang University, Hangzhou, Zhejiang 310009, P.R. China

**Keywords:** N-acetylcysteine, spinal cord trauma, mitochondrial dysfunction, oxidative stress, neuroprotection, locomotor function

## Abstract

Following spinal cord trauma, mitochondrial dysfunction associated with increased oxidative stress is a critical event leading to leukocyte inflammatory responses, neuronal cell death and demyelination, contributing to permanent locomotor and neurological disability. The present study demonstrated that the mitochondrial enhancer N-acetylcysteine (NAC) may restore redox balance via enhancement of mitochondrial respiratory activity following traumatic spinal cord injury (SCI). In addition, NAC ameliorates oxidative stress-induced neuronal loss, demyelination, leukocyte infiltration and inflammatory mediator expression and improves long-term locomotor function. Furthermore, neuronal survival and neurological recovery are significantly correlated with increased mitochondrial bioenergetics in SCI following treatment with NAC. Therefore, NAC may represent a potential therapeutic agent for preserving mitochondrial dynamics and integrity following traumatic SCI.

## Introduction

Oxidative stress contributes to a cascade of secondary damage following spinal cord injury (SCI), which results in inflammatory cell infiltration, neuronal and glial cell destruction, neuronal dysfunction and cell death (1). It has been demonstrated that mitochondrial dysfunction is a principle source of increased oxidative stress following SCI (2–4). Pharmacological treatments have demonstrated that mitochondria are important as a therapeutic target to promote neuronal recovery and regeneration against oxidative SCI (3,5). Thus, preventing mitochondrial dysfunction and stabilizing mitochondrial integrity may be considered as a potential approach for antioxidant based interventions following traumatic SCI.

The pathophysiological antioxidative defense mechanisms against mitochondrial dysfunction and reactive oxygen species (ROS) production in the injured spinal cord are complicated (3–7). N-acetylcysteine (NAC), a derivative of cysteine, acts as a mitochondrial enhancer, which has a broad range of activities, including anti-inflammatory and antioxidant effects in neuronal disorders associated with excessive free radical production and oxidative tissue damage (8,9). Previous studies have demonstrated that acute NAC administration reduced neuronal degeneration and attenuated the microglia and inflammatory responses following SCI in rodent models (9,10). However, whether NAC has neuroprotective effects on long-term neurological recovery in the injured spinal cord following traumatic SCI remains to be elucidated. The aim of the present study was to evaluate the effects of NAC on spinal cord trauma.

## Materials and methods

### SCI

Adult female C57BL/6 mice weighing 18–22 g were anesthetized intraperitoneally with sodium pentobarbital (40 mg/kg) and subjected to a moderate spinal cord contusion trauma. A laminectomy was performed at the T9 level and the exposed dorsal surface of the spinal cord was subjected to moderate contusion injury as described previously (11). Following SCI, the skin was closed with wound clips. Animal body temperature was maintained at 37°C with a warming blanket throughout the surgery and during the recovery from anesthesia. For the sham-surgery controls (SHAM), the animals underwent a T9 laminectomy without contusion injury. Surgical interventions and postoperative animal care were conducted in accordance with the National Institutes of Health Guide for the Care and Use of Laboratory Animals (National Institutes of Health, Bethesda, MD, USA) and with approval from the Animal Subjects Committee at Zhejiang University (Hangzhou, China). The study was approved by the ethics committee of Zhejiang University (Hangzhou, China).

### Drug treatment

NAC dissolved in phosphate-buffered saline (PBS; Sigma-Aldrich, St. Louis, MO, USA) was administered to corresponding SHAM and SCI mice (SHAM+NAC and SCI+NAC) via intraperitoneal injection (100 mg/kg) (12–15) 1 day prior to SCI and then once a day for 28 days for behavioral assessment or for the time-points indicated on the corresponding figures for other experiments following SCI. The current dose of NAC administration was applied on the basis of mitochondrial function restoration in our experiments. Sterile saline for vehicle control (VEH) was administered into corresponding SHAM and SCI mice (SHAM+VEH and SCI+VEH). Significant side effects resulting from NAC treatment, including alterations in body weight or an increase in the mortality rate, were not observed during the present experiments.

### Mitochondrial integrity and function

Mitochondrial isolation was performed as described previously with the following modifications (3–7). The final mitochondria was stored on ice during assessments of mitochondrial function. Mitochondrial respiration and enzyme activity was assessed using a miniature Clark-type electrode following procedures described previously (3–7). The oxidative substrates and inhibitors of different enzyme complexes of the mitochondrial electron transport system were added to assess mitochondrial respiration. A total of three independent oxymetric traces for each sample were obtained and the respiration rates averaged.

### Histology and immunohistochemistry

Histological and immunohistochemistry procedures were performed as previously described with certain modifications (16). Spinal cord tissues were transferred to a 25% sucrose solution prior to processing for histological analysis. The spinal cords were subsequently frozen, sectioned at 7 *μ*m thickness and stained with hematoxylin and eosin for detection of spinal cord injuries and leukocyte infiltration. A scale of 0–4 represented the severity of spinal cord injury: 0, none or minor; 1, modest or limited; 2, intermediate; 3, widespread or prominent and 4, widespread and most prominent. These tasks were conducted in a blinded manner.

To assess white matter sparing and neuronal survival, the serial 7 *μ*m transverse sections at 250 *μ*m intervals surrounding the epicenter of the lesion 28 days after SCI were stained with Nissl for neurons and Luxol fast blue for myelin. A total of 13 sequential sections spanning 3,000 *μ*m of spinal cord length, which included six sections, rostral and caudal to the section at the epicenter, were assessed in SHAM and SCI mice. The images of Nissl and Luxol fast blue staining were analyzed using Image J software version 1.48 (National Institutes of Health).

Non-heme iron was analyzed using Perls’ prussian blue to assess iron deposition in the spinal cord. Quantitative measurement of non-heme iron was performed on spinal cord tissue as previously described with a number of modifications (17). Integrated density of non-heme iron accumulation was determined using Image J software (National Institutes of Health).

For immunofluorescence staining, the transverse sections 250 *μ*m caudal and rostral to the epicenter of the lesion (T10) 28 days after SCI were used. The spinal sections were washed in PBS for 10 min, followed by washing with PBS containing 0.3% Tween 20 (Sigma-Aldrich) for 10 min and blocked with blocking serum for 2 h. The sections were incubated with mouse anti-neuronal nuclei (NeuN; 1:100; Santa Cruz Biotechnology, Inc., Santa Cruz, CA, USA) diluted in PBS overnight at 4°C. Nuclei were stained for 10 min at room temperature with 300 ng/ml DAPI (Sigma-Aldrich). Images were acquired using a Leica DM LA upright microscope (Leica, Mannheim, Germany) and were analyzed using Image J software by three investigators.

### Myeloperoxidase (MPO) activity assay

MPO activity, as an indicator of leukocyte infiltration was performed in spinal cord tissue as previously described (18). The MPO activity was defined as the quantity of enzyme required to degrade 1 mmol H_2_O_2_ per minute at 37°C, expressed as units of MPO/g wet tissue.

### Reverse transcription-quantitative polymerase chain reaction (RT-qPCR)

Total RNA was prepared with TRIzol reagent (Invitrogen Life Technologies, Carlsbad, CA, USA). Complementary DNA synthesis and RT-qPCR were performed using a previously described method (11). The primer sequences used were as follows: Tumor necrosis factor-α (TNF-α), forward 5′-CCCAGACCCTCACACTCAGAT-3′ and reverse 5′-TTGTCCCTTGAAGAGAACCTG-3′; interleukin (IL)-1β, forward 5′-GCAGCTACCTATGTCTTGCCCGTG-3′ and reverse 5′-GTCGTTGCTTGTCTCTCCTTGTA-3′; IL-6, forward 5′-AAGTTTCTCTCCGCAAGATACTTCCAGCCA-3′ and reverse 5′-AGGCAAATTTCCTGGTTATATCCAGTT-3′; inducible nitric oxide synthase (iNOS), forward 5′-CTCCATGACTCTCAGCACAGAG-3′ and reverse 5′-GCACCGAAGATATCCTCATGAT-3′.

### ELISA

To determine the cytokine and ROS levels, the lesion site was rapidly dissected and homogenized in PBS 24 h after SCI. Following centrifugation at 4°C for 15 min at 900 × g, the supernatants were used to measure the concentrations of IL-6, IL-1β, TNF-α (R&D Systems, Minneapolis, MN, USA), iNOS, glutathione peroxidase (GSH-Px) and superoxide dismutase (SOD; Cusabio Biotech Co., Ltd., Wuhan, China) using corresponding ELISA kits.

### Western blot analysis

A 0.5 cm length of cord, centered over the site of impact and representing the epicenter, was lysed on ice for 30 min with 50 mM Tris-HCl (pH 7.5), 150 mM NaCl, 1 mM EDTA, 1 mM EGTA, 25 mM NaF, 5 mM sodium pyrophosphate, 1 mM Na_3_VO_4_ and protease inhibitors (Roche Diagnostics GmbH, Mannheim, Germany). Cell lysates were clarified by centrifugation at 10,400 × g for 15 min at 4°C and the supernatants were collected and assayed for protein concentration using a bicinchoninic acid protein assay kit (Pierce Biotechnology, Inc., Rockford, IL, USA). Immunoblot analysis was performed as described previously (11). The following antibodies were used: Rabbit polyclonal anti-heme oxygenase-1 (HO-1; 1:200; cat no. sc-10789; Santa Cruz Biotechnology, Inc.) and rabbit polyclonal anti-α/β-Tubulin (1:1,000; cat no. 2148; Cell Signaling Technology, Inc., Danvers, MA, USA). Tubulin served as a loading control. Subsequently, the membrane was incubated with goat anti-rabbit Alexa Fluor 488 (Jackson Immunoresearch, West Grove, PA, USA). at a concentration of 1:200. The immunoblot analysis was repeated three times and the band density of immunoblots obtained using Image J software were subjected to statistical analysis.

### Behavioral analysis

The behavioral test was scored in accordance with the rules of Basso, Beattie and Bresnahan (BBB), which comprise 21 criteria for the movement of lower limbs, from complete paralysis to complete mobility (19). These criteria are based on the accurate observation of the lower limbs, including movement, step and coordinated motor action. This rating scale takes into account limb movement, stepping, coordination and trunk stability. Mice were assessed at 1 and 3 days and weekly thereafter until euthanasia 28 days after SCI. Motor performance on a rotarod was also evaluated as well as the ability to traverse a wire grid (17), in sequence, at 26, 27 and 28 days after SCI. There were three trials daily, with a total of nine trials for each test. In each of these tests, the mean score from each mouse was used.

### Statistical analysis

Statistical analysis was performed using GraphPad Prism software version 6 (GraphPad Software, Inc., La Jolla, CA, USA). P<0.05 was considered to indicate a statistically significant difference. Data are presented as the mean ± standard error of the mean of three independent experiments.

## Results

### Effects of NAC on mitochondrial dysfunction-induced oxidative stress

**Figure 1:**
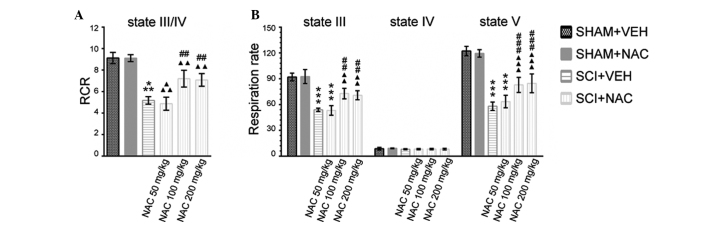
Effects of NAC on mitochondrial function and integrity. Data are presented as the mean ± standard error of the mean of three independent experiments (n=3 per group). ^***^P<0.001, SCI+VEH mice versus SHAM+VEH mice; ^##^P<0.01, ^###^P<0.001, SCI+NAC mice versus SCI+VEH mice; ^ΔΔ^P<0.01, ^ΔΔΔ^P<0.001, SCI+NAC mice versus SHAM+NAC mice. (A) RCR as a measure of mitochondrial integrity was calculated as the ratio of state III/state IV slopes at 24 h in SHAM and SCI mice treated with NAC or VEH. (B) Mitochondrial respiration rates were calculated as nmols oxygen/min/mg protein at 24 h in SHAM and SCI mice treated with NAC or VEH. NAC, N-acetylcysteine; VEH, vehicle; SCI, spinal cord injury; RCR, respiratory control ratio.

Mitochondrial dysfunction is critical in the progression of secondary injury in SCI (4,7,20). To assess whether mitochondrial integrity or function were directly affected by SCI, alterations in the mitochondrial respiratory control ratio (RCR) and respiration rates were analyzed in the injured spinal cord (Fig. 1A and B). A significant decrease in RCR and state III and state V-complex I respiration in SCI+VEH mice compared with SHAM mice was observed 24 h after SCI. NAC treatment at 50 mg/kg/day had no effect on a lower mitochondrial respiratory capacity caused by SCI. When NAC treatment in SCI mice was at dosages of 100 or 200 mg/kg per day, a significant restoration of mitochondrial RCR and respiration rates was observed compared with VEH treatment in SCI mice, although the RCR and respiration rates in SCI+NAC mice remained significantly lower than SHAM mice. No significant differences were identified in improved mitochondrial respiratory function between SCI+NAC 100 mg/kg/day mice and SCI+NAC 200 mg/kg/day mice.

**Figure 2:**
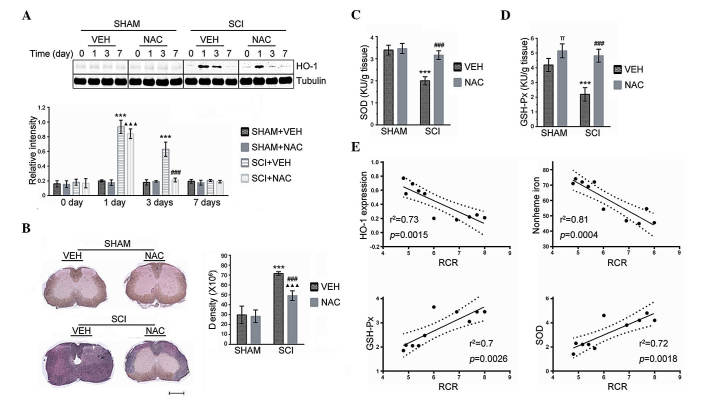
Effects of NAC on oxidative stress. Data are presented as the mean ± standard error of the mean of three independent experiments (n=3 per group) ^***^P<0.001, SCI+VEH mice versus SHAM+VEH mice; ^###^P<0.001, SCI+NAC mice versus SCI+VEH mice; ^ΔΔΔ^P<0.001, SCI+NAC mice versus SHAM+NAC mice; ^π^P<0.05, SHAM+NAC mice versus SHAM+VEH mice. (A) HO-1 expression by quantitative immunoblot analysis was performed at the indicated time points in SHAM and SCI mice treated with NAC or VEH. (B) Modified Perl’s staining was performed to detect non-heme iron in SHAM and SCI mice treated with NAC or VEH at 14 days. Scale bar indicates 500 *μ*m. Accumulation of non-heme iron detected by Perl’s staining was analyzed using NIH Image J and expressed as integrated density measurements. (C) ELISA of SOD was performed at 24 h in SHAM and SCI mice treated with NAC or VEH. (D) ELISA of GSH-Px was performed at 24 h in SHAM and SCI mice treated with NAC or VEH. (E) Correlation between mitochondrial RCR and oxidative stress in injured spinal cord. NAC, N-Acetyl-Cysteine; VEH, vehicle; SCI, spinal cord injury; RCR, respiratory control ratio; SOD, superoxide dismutase; GSH-Px, glutathione peroxidase; HO-1, heme oxygenase-1.

The effects of NAC on the oxidant and antioxidant status induced by mitochondrial dysfunction in SCI were subsequently evaluated. Indicators of oxidative stress, including GSH-Px, HO-1, non-heme iron and SOD were measured in all SHAM and SCI mice. Analysis of immunoblots revealed an elevation of HO-1 in SCI mice treated with VEH or with NAC (100 mg/kg/day) at 24 h compared with SHAM mice (Fig. 2A). However, the expression of HO-1 was significantly decreased at 3 days in SCI mice treated with NAC, but not in those treated with VEH. No significant difference was identified in HO-1 protein expression between SCI and SHAM mice at 7 days. In addition, accumulation of non-heme iron was reduced in the injured spinal cord at 14 days (Fig. 2B) in SCI mice treated with NAC (100 mg/kg/day). The GSH-Px and SOD activity in SCI+VEH mice was significantly lower than SHAM mice, whereas NAC (100 mg/kg/day) significantly rescued the GSH-Px and SOD activities in mice at 24 h after SCI (Fig. 2C and D). Furthermore, significant positive correlations were observed between the higher GSH-Px/SOD activities and increased mitochondrial RCR (Fig. 2E). By contrast, negative correlations were identified between HO-1 expression/non-heme iron accumulation and mitochondrial RCR (Fig. 2E), indicating that mitochondrial respiratory dysfunction is directly linked to oxidative stress-induced redox imbalance in SCI.

### Effects of NAC on histological alterations and neuronal survival

**Figure 3:**
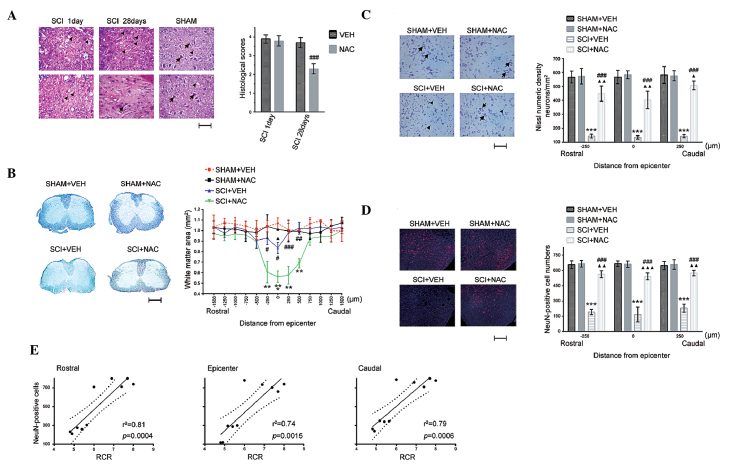
Effects of NAC on histological changes. Data are presented as the mean ± standard error of the mean of three independent experiments (n=3 per group). ^**^P<0.01, ^***^P<0.001, SCI+VEH mice versus SHAM+VEH mice; ^#^P<0.05, ^##^P<0.01, ^###^P<0.001, SCI+NAC mice versus SCI+VEH mice; ^Δ^P<0.05, ^ΔΔ^P<0.01, ^ΔΔΔ^P<0.001, SCI+NAC mice versus SHAM+VEH mice. (A) SCI and leukocyte infiltration by hematoxylin and eosin staining at 1 day and 28 days in SHAM and SCI mice treated with NAC or VEH (scale bar=100 *μ*m). Arrows indicate normal neurons; triangles indicate glia. Histological scores were measured for the SCI severity at 1 and 28 days in SCI mice treated with NAC or VEH. (B) White matter sparing by Luxol fast blue staining at 28 days in SHAM and SCI mice treated with NAC or VEH (scale bar=500 *μ*m). Data are expressed as the proportional area. (C) Quantification of Nissl numeric density (neurons/mm^2^) at 28 days in SHAM and SCI mice treated with NAC or VEH (scale bar=100 *μ*m). Arrows indicate normal neurons; triangles indicate neuron death. (D) Quantification of immunofluorescence staining of NeuN (red) and 4′,6-diamidino-2-phenylindole (blue) at 28 days in SHAM and SCI mice treated with NAC or VEH (scale bar=100 *μ*m). (E) Correlation between mitochondrial RCR and neuronal survival in SCI. NAC, N-acetylcysteine; VEH, vehicle; SCI, spinal cord injury; RCR, respiratory control ratio; NeuN, neuronal nuclei.

Subsequently, the anti-oxidative protection of NAC on spinal cord histological and neurological alterations following SCI was examined. There were homogenous and intact tissue structures in uninjured spinal cord in all SHAM mice, whereas SCI caused mechanical insults, including edema, hemorrhage, leukocyte recruitment and loosened tissue structure in all SCI mice on day 1 (Fig. 3A). Compared with SCI+VEH mice, morphological changes and inflammatory cell infiltration were significantly alleviated in SCI mice treated with NAC (100 mg/kg/day) at 28 days. Subsequently, it was investigated whether NAC repressed demyelination following SCI. White matter sparing was compared between SHAM and SCI mice using Luxol fast blue staining (Fig. 3B). Similarly, white matter sparing at the epicenter (T10) was significantly decreased in SCI+VEH mice compared with SCI+NAC mice at 28 days. Statistically significant differences were identified at the epicenter and at rostral (250 *μ*m) and caudal (250, 500 and 750 *μ*m) sites between SCI+VEH and SCI+NAC mice.

To identify whether NAC treatment (100 mg/kg/day) suppressed neuronal death in SCI, the protective effects of NAC on neuronal survival was examined using Nissl and NeuN staining in SHAM and SCI mice (Fig. 3C and D). No significant difference was identified in Nissl-stained neurons and NeuN-positive cell numbers in the spinal cord between SHAM+VEH mice and SHAM+NAC mice, whereas the number of Nissl-stained neurons and NeuN-positive cells in SCI+NAC mice were higher than that in SCI+VEH mice at the epicenter and at rostral (250 *μ*m) and caudal (250 *μ*m) sites at 28 days. In addition, mitochondrial RCR is positively correlated with neuronal survival (NeuN-positive cell numbers) in the injured spinal cord. These results suggest that recovery of mitochondrial function using NAC may promote histological improvement and neuronal survival following SCI.

### Effects of NAC on leukocyte inflammatory responses

**Figure 4:**
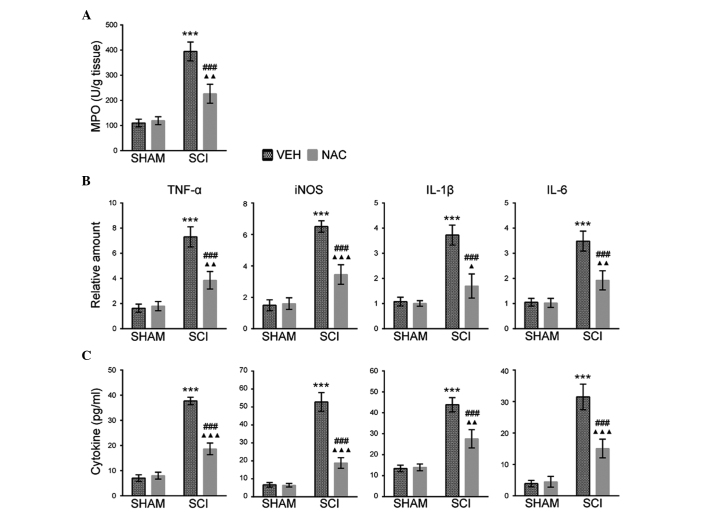
Effects of NAC on MPO activity and inflammatory mediator expression. Data are presented as the mean ± standard error of the mean of three independent experiments (n=3 per group). ^***^P<0.001, SCI+VEH mice versus SHAM+VEH mice; ^###^P<0.001, SCI+NAC mice versus SCI+VEH mice; ^Δ^P<0.05, ^ΔΔ^P<0.01, ^ΔΔΔ^P<0.001, SCI+NAC mice versus SHAM+NAC mice. (A) MPO activity as an indicator of leukocyte infiltration was analyzed at 24 h in SHAM and SCI mice treated with NAC or VEH. (B) Reverse transcription-quantitative polymerase chain reaction of TNF-α, IL-1β (at 2 h), IL-6 and iNOS (at 6 h) was performed in SHAM and SCI mice treated with NAC or VEH. (C) ELISA of TNF-α, IL-1β, IL-6 and iNOS was performed at 24 h in SHAM and SCI mice treated with NAC or VEH. NAC, N-acetylcysteine; VEH, vehicle; SCI, spinal cord injury; RCR, respiratory control ratio; TNF-α, tumor necrosis factor-α; IL, interleukin; MPO, myeloperoxidase; iNOS, inducible nitric oxide synthase.

The histological changes of SCI were associated with leukocyte and inflammatory mediator accumulation in the injured spinal cord of mice (16). Therefore, the effects of NAC (100 mg/kg/day) on neutrophil infiltration were investigated using an MPO activity assay in SHAM and SCI mice at 1 day. The MPO activity was higher in SCI mice treated with VEH than those treated with NAC (Fig. 4A). The effects of NAC treatment on the expression of inflammatory mediators were also examined, including IL-1β, IL-6, iNOS and TNF-α by RT-qPCR and ELISA assays in SHAM and SCI mice at the indicated time points. It was identified that the mRNA expression levels of IL-1β, TNF-α (at 2 h), IL-6 and iNOS (at 6 h) were increased in SCI mice treated with VEH compared with mice treated with NAC, or than all SHAM mice and SCI mice (Fig. 4B). ELISA assays also revealed similar expression levels of inflammatory mediators at 24 h in SHAM and SCI mice (Fig. 4C). This suggests that NAC may inhibit leukocyte infiltration and inflammatory responses in SCI mice.

### Effects of NAC on long-term functional recovery

**Figure 5:**
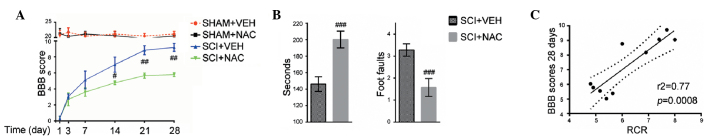
Effects of NAC on long-term functional recovery. Data are presented as the mean ± standard error of the mean of three independent experiments (n=10 per group). ^#^P<0.05, ^##^P<0.01, ^###^P<0.001, SCI+NAC mice versus SCI+VEH mice. (A) Functional recovery as determined by the BBB scale in SHAM and SCI mice treated with NAC or VEH at the indicated time points. (B) Performance on a rotarod and grid walking in SCI mice treated with VEH or NAC. (C) Correlation between mitochondrial RCR and locomotor recovery following SCI. NAC, N-acetylcysteine; VEH, vehicle; SCI, spinal cord injury; RCR, respiratory control ratio; BBB, Basso, Beattie and Bresnahan.

In order to evaluate whether the neuroprotective effects of NAC (100 mg/kg/day) were associated with long-term functional recovery, the BBB, rotarod and grid-walk test scores were measured for 28 days. It was observed that hind-limb movements were eradicated immediately following SCI compared with SHAM mice as assessed using the BBB scale. The BBB scores in all SCI mice increased after 1 day. The averages of the BBB scores were higher in SCI mice treated with NAC than in SCI+VEH mice from days 14–28 (Fig. 5A). Subsequent assessment of rotarod and grid-walk performance (Fig. 5B) revealed similar locomotor functional improvements in SCI mice treated with NAC, relative to their respective controls, thereby confirming that NAC treatment may enhance long-term recovery of locomotor function in SCI mice. A significant positive correlation was observed between the higher BBB scores (28 days) and increased mitochondrial RCR in SCI mice treated with NAC (Fig. 5C). These results indicate that mitochondrial function improved by NAC is beneficial for long-term locomotor recovery in SCI mice.

## Discussion

Oxidative stress contributes to several pathological responses following SCI, including leukocyte infiltration, demyelination and neuronal death that exacerbate spinal cord damage and impair neurological recovery (4,7,20,21). Suppression of oxidative damage in the spinal cord has been verified to ameliorate the neurological dysfunction following spinal cord trauma (16,17,21). In the present study, it was demonstrated that the mitochondrial enhancer NAC exerted antioxidant and neuroprotective effects in SCI mice. The administration of NAC in SCI mice suppressed oxidative stress, attenuated histopathological alterations, the degeneration of injured spinal cord and the reduction of neuronal loss, inhibited the expression of proinflammatory factors, preserved white matter area and promoted locomotor functional recovery. These results clearly suggest that NAC is an effective therapy for the treatment of traumatic SCI.

Spinal cord trauma initially causes immediate, primarily mechanical neuronal and vascular tissue damage followed by secondary neural dysfunction and neurodegeneration in the injured spinal cord (1). There were multiple independent, however, interrelated, biochemical and pathophysiological pathways underlying the secondary damage following SCI (7,10). It is apparent that oxidative stress is one of the principle pathogenic pathways involved in SCI (7,10). Previous studies have demonstrated that increased oxidative damage was primarily associated with mitochondrial dysfunction in the injured spinal cord (2–4). Thus, the regulation of oxidative stress through pharmacological targeting of mitochondrial dysfunction may provide critical neuroprotection for traumatic SCI. In the present study, it was revealed that SCI severely impairs mitochondrial function and integrity in the injured spinal cord of mice and acute NAC treatment at 100 mg/kg concentration was able to significantly preserve mitochondrial dynamics and integrity. Correlation analyses between neurological recoveries and mitochondrial respiratory activities also provided marked evidence that improved mitochondrial oxidative function with NAC treatment following spinal cord trauma was a direct result of greater neuronal survival and locomotor response.

The present study assessed whether NAC conspicuously decreased oxidative stress and restored redox state balance in SCI mice. These results are consistent with previous findings that excessive oxidative stress following SCI results in decreased activities of key mitochondrial respiratory enzymes and NAC is also reported to directly improve mitochondrial energy production and stabilize mitochondrial membranes (3–7). It was also observed that NAC treatment rapidly inhibited HO-1 expression, which acts as an antioxidant and neuroprotective effector molecule in the injured spinal cord (21). These results may be explained by the suppression of the nuclear erythroid 2-related factor 2 (NRF2)/antioxidant response elements pathway by NAC (22) and NRF2 may directly regulate HO-1 promoter activity (23). However, the potential disadvantageous function of NAC may be counterbalanced by its beneficial effects on maintaining mitochondrial function and integrity following SCI (7). In addition, NAC may enhance neuronal glutathione levels through the extracellular signal-regulated kinase (ERK)/eukaryotic initiation factor 2/activating transcription factor 4 pathway, which reduces oxidant-induced neuronal injury and cell death and scavenges free radical species in the injured spinal cord (24).

It is well established that traumatic SCI results in neuronal loss and degeneration (7,10,21). Spinal cord trauma also leads to the delayed retrograde reaction in spinal motor and sensory neurons (25,26). The present study confirmed previous observations that traumatic SCI induces spinal neuron degeneration, spinal cord dysfunction and associated locomotor deficits, whereas NAC treatment exhibited marked neuroprotective effects on lesioned spinal neurons, including larger areas of white matter sparing and less neuronal death in SCI mice. Consistent with previous studies (8,9), NAC treatment may immediately suppress leukocyte infiltration and the expression of several important neuroinflammatory mediators, including IL-1β, IL-6, iNOS and TNF-α in SCI mice. Furthermore, NAC significantly improved hind limb function within the first week of administration and promoted long-term locomotor recovery following SCI. The mechanisms underlying the effects of NAC on recovery of the injured spinal cord neurons and locomotor function following traumatic SCI is at least partially dependent upon the ability of NAC to positively regulate the neurotrophic factor activated Ras/ERK pathway. These neurotrophic factors, including brain-derived neurotrophic factor, nerve growth factor and glial cell line-derived neurotrophic factor are essential to maintain neuronal survival and neuronal structure (27–29). Accordingly, the current findings indicate that neuroprotection of spinal cord neurons following NAC treatment after SCI has a major impact on long-term biochemical, histological and neurological recovery.

Taken together, the neuroprotective effects of NAC on oxidative stress and mitochondrial integrity following traumatic SCI have been demonstrated. The present study revealed that NAC suppressed excessive oxidative stress, attenuated mitochondrial dysfunction and inflammatory responses, promoted white matter sparing and improved long-term neuronal survival and neurological recovery in SCI mice. The current results suggest that NAC may provide potential therapeutic applications for preventing mitochondrial dysfunction-induced oxidative stress following spinal cord trauma.
